# EEG-Based BCI Emotion Recognition: A Survey

**DOI:** 10.3390/s20185083

**Published:** 2020-09-07

**Authors:** Edgar P. Torres, Edgar A. Torres, Myriam Hernández-Álvarez, Sang Guun Yoo

**Affiliations:** 1 Escuela Politécnica Nacional, Facultad de Ingeniería de Sistemas, Departamento de Informática y Ciencias de la Computación, Quito 170143, Ecuador; edgar.torres@epn.edu.ec (E.P.T.); sang.yoo@epn.edu.ec (S.G.Y.); 2 Pontificia Universidad Católica del Ecuador; Quito 170143, Ecuador; etorresh777@gmail.com

**Keywords:** emotion recognition, emotion elicitation, dataset, emotion representation, feature selection, feature extraction, classification, computer science, artificial intelligence, affective computing

## Abstract

Affecting computing is an artificial intelligence area of study that recognizes, interprets, processes, and simulates human affects. The user’s emotional states can be sensed through electroencephalography (EEG)-based Brain Computer Interfaces (BCI) devices. Research in emotion recognition using these tools is a rapidly growing field with multiple inter-disciplinary applications. This article performs a survey of the pertinent scientific literature from 2015 to 2020. It presents trends and a comparative analysis of algorithm applications in new implementations from a computer science perspective. Our survey gives an overview of datasets, emotion elicitation methods, feature extraction and selection, classification algorithms, and performance evaluation. Lastly, we provide insights for future developments.

## 1. Introduction

Affective computing is a branch of artificial intelligence. It is computing that relates to, arises from, or influences emotions [1]. Automatic emotion recognition is an area of study that forms part of affective computing. Research in this area is rapidly evolving thanks to the availability of affordable devices for capturing brain signals, which serve as inputs for systems that decode the relationship between emotions and electroencephalographic (EEG) variations. These devices are called EEG-based brain-computer interfaces (BCIs).

Affective states play an essential role in decision-making. Such states can facilitate or hinder problem-solving. Emotion recognition takes advantage of positive affective states, enhances emotional intelligence, and consequently improves professional and personal success [2]. Moreover, emotion self-awareness can help people manage their mental health and optimize their work performance. Automatic systems can increase our understanding of emotions, and therefore promote effective communication among individuals and human-to-machine information exchanges. Automatic EEG-based emotion recognition could also help enrich people’s relationships with their environment. Besides, automatic emotion recognition will play an essential role in artificial intelligence entities designed for human interaction [3].

According to Gartner’s 2019 Hype Cycle report on trending research topics, affective computing is at the innovation trigger stage, which is evidenced by the field’s copious publications. However, there are still no defined standards for the different components of the systems that recognize emotions using EEG signals, and it is still challenging to detect and classify emotions reliably. Thus, a survey that updates the information in the emotion recognition field, with a focus on new computational developments, is worthwhile.

This work reviews emotion recognition advances using EEG signals and BCI to (1) identify trends in algorithm usage and technology, (2) detect potential errors that must be overcome for better results, and (3) identify possible knowledge gaps in the field. The aim is to distinguish what has already been done in systems implementations and catch a glimpse of what could lie ahead. For context, our study is a survey from 2015 to 2020.

The present article gives an overview of datasets, emotion elicitation methods, feature extraction and selection, classification algorithms, and in general terms, computer intelligence techniques used in this field. We present a brief review of the components of an EEG-based system to recognize emotions and highlight trends showing statistics of their use in the literature. We deliver a compilation of papers describing new implementations, analyzing their inputs, tools, and considered classes. This up-to-date information could be used to discover and suggest new research paths.

The present survey followed the guidelines of [4]. We used Semanticscholar.org for searches of sources because it links to the major databases that contain journals and conferences proceedings. The search criteria were the keywords linked to our review’s objectives.

We extracted articles from journals and conferences that present new implementations of computational intelligence techniques. Concretely, the analyzed papers’ primary objectives were computational systems that applied algorithms for the detection and classification of emotions using EEG-based BCI devices. Such studies also included performance measures that allowed a comparison of results while taking into account the classified number of emotions.

As a result, we obtained 136 journal articles, 63 conference papers, and 15 reviews. Each whole article was read to have complete information to guide the application of inclusion and exclusion filters. The inclusion criteria were: (1) The articles were published in the considered period in peer-reviewed journals and conferences, (2) they constitute emotion recognition systems that used EEG-based BCI devices with a focus on computational intelligence applications, and (3) they include experimental setups and performance evaluations. Lastly, we applied additional exclusion criteria and eliminated review articles and other studies that have a different perspective as medical studies for diagnosis or assessment.

With these considerations, we selected 36 journal studies and 24 conference papers. From this group, we extracted statistical data about computational techniques to detect trends and perform a comparative analysis. Finally, from these 60 papers, we chose a sample of 31 articles to show a summary of technical details, components, and algorithms. It should be noted that according to generally accepted practices, 31 observations are sufficient for statistically valid conclusions due to the central limit theorem. Then, from this subsample of articles, we obtained some additional data and tendencies.

This document is organized as follows: Section 1 presents an introduction of the topic, with an overview of BCI devices, emotion representations, and correlations among brain locations, frequency bands, and affective states. Section 2 shows the structure of EEG-based BCI systems for emotion recognition. Their principal components are revised: (1) Signal acquisition, (2) preprocessing, (3) feature extraction, (4) feature selection, (5) classification, and (6) performance evaluation. Section 3 analyzes the components of our chosen research pieces and discusses trends and challenges. Section 4 presents future work. Section 5 features the conclusions or this survey.

### 1.1. EEG-Based BCI in Emotion Recognition

Many studies suggest that emotional states are associated with electrical activity that is produced in the central nervous system. Brain activity can be detected through its electrical signals by sensing its variations, locations, and functional interactions [5] using EEG devices. EEG signals have excellent temporal resolution and are a direct measurement of neuronal activity. These signals cannot be manipulated or simulated to fake an emotional state, so they provide reliable information. The challenge is to decode this information and map it to specific emotions.

One affordable and convenient way to detect EEG signals is through EEG-based BCI devices that are non-invasive, low cost, and even wearable, such as helmets and headbands. The development of these tools has facilitated the emergence of abundant research in the emotion recognition field. 

Some scientists predict that EEG-based BCI devices will soon improve their usability. Therefore, shortly, they could be used on an everyday basis for emotion detection with several purposes, such as emotion monitoring in health care facilities, gaming and entertainment, teaching-learning scenarios, and for optimizing performance in the workplace [6], among other applications.

### 1.2. Emotion Representations

Emotions can be represented using different general models [7]. The most used are the discrete model and the dimensional models. The discrete model identifies basic, innate, and universal emotions from which all other emotions can be derived. Some authors state that these primary emotions are happiness, sadness, anger, surprise, disgust, and fear [8]. Some researchers consider that this model has limitations to represent specific emotions in a broader range of affective states. 

Alternatively, dimensional models can express complex emotions in a two-dimensional continuous space: Valence-arousal (VA), or in three dimensions: Valence, arousal, and dominance (VAD) [9]. The VA model has valence and arousal as axes. Valence is used to rate positive and negative emotions and ranges from happy to unhappy (or sad). Arousal measures emotions from calm to stimulated (or excited). Three-dimensional models add a dominance axis to evaluate from submissive (powerless) to empowered emotions. This representation distinguishes emotions that are jointly represented in the VA model. For instance, fear and anger have similar valence-arousal representations on the VA plane. Thus, three-dimensional models improve “emotional resolution” through the dominance dimension. In this example, fear is a submissive feeling, but anger requires power [10]. Hence, the dominance dimension improves the differentiation between these two emotions.

**Figure 1:**
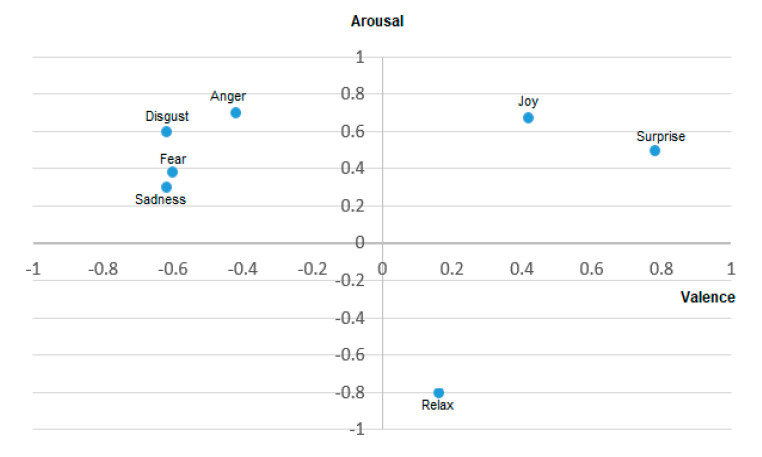
Emotional states in the Valence-Arousal space [11].

**Figure 2:**
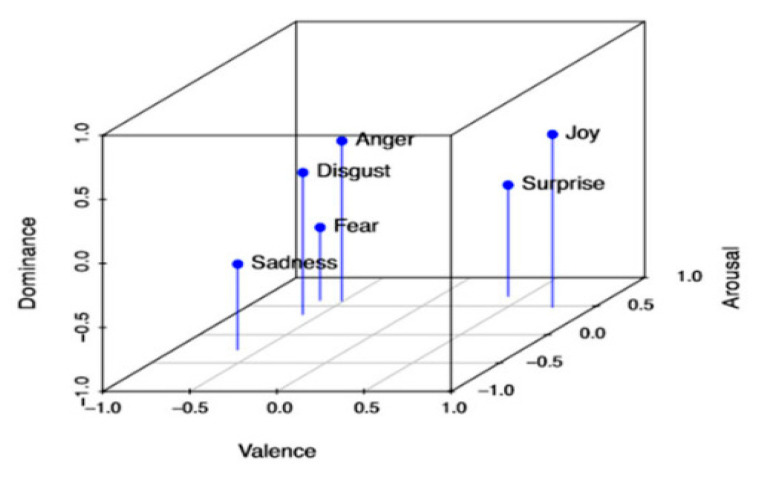
Emotional states in the Valence-Arousal-Dominance space [12].

Figure 1 shows a VA plane with the representation of basic emotions. The horizontal axis corresponds to valence dimensions, from positive to negative emotions. Likewise, the vertical axis corresponds to arousal. These two variables can be thought of as emotional state components [5]. Figure 2 presents the VAD space with a representation of the same basic emotions.

Table 1 shows that some researchers studying EEG-based functional connectivity in the brain have reported a relationship between specific brain areas and emotional states. Studies that take at-single-electrode-level analysis into account have shown that asymmetric activity at the frontal site in the alpha band is associated with emotion. Ekman and Davidson found that enjoyment generated an activation of the brain’s left frontal parts [13]. Another study found a left frontal activity reduction when volunteers adopted fear expressions [14]. Increased power in theta bands at the frontal midline is associated with pleasurable emotions, and the opposite has been observed with unpleasant feelings [15].

Several studies confirm that frequency bands are related to affective responses. However, emotions are complex processes. The authors in [15] assert that the recognition of different emotional states may be more valid if EEG-based functional connectivity is examined, rather than a single analysis at the electrode level. Correlation, coherence, and phase synchronization indices between EEG electrode pairs are used to estimate functional connectivity between different brain locations. Likewise, differential entropy (DE), and its derivatives like differential asymmetry (DASM), rational asymmetry (RASM), and differential caudality (DCAU) measure functional dissimilarities. Such features are calculated through logarithmic power spectral density for a fixed-length EEG sequence, plus the differences and ratios between DE features of hemispheric asymmetry electrodes [19].

The growing consensus seems to be that a simple mapping between emotions and specific brain structures is inconsistent with observations of different emotions activating the same structure, or one emotion activating several structures [20]. Additionally, functional connectivity between brain regions or signal complexity measures may help to detect and describe emotional states [21].

## 2. EEG-Based BCI Systems for Emotion Recognition

**Figure 3:**
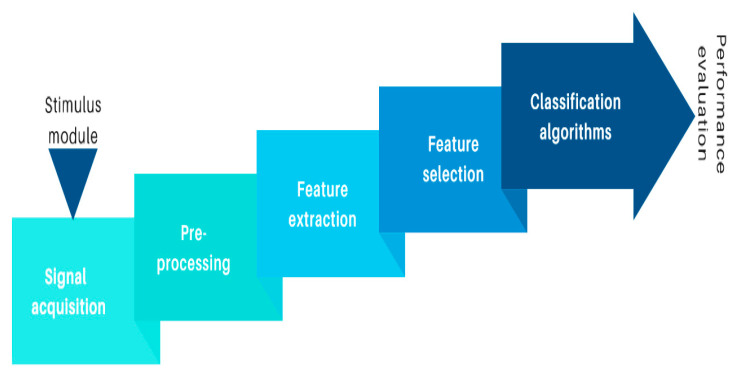
Components of an EEG-based BCI for emotion recognition.

Figure 3 presents the structure of an EEG-based BCI system for emotion recognition. The processes of signal acquisition, preprocessing, feature extraction, feature selection, classification, and performance evaluation can be distinguished and will be reviewed in the following subsections.

### 2.1. Signal Acquisition

Inexpensive wearable EEG helmets and headsets that position noninvasive electrodes along the scalp can efficiently acquire EEG signals. The clinical definition of EEG is an electrical signal recording of brain activity over time. Thus, electrodes capture signals, amplify them, and send them to a computer (or mobile device) for storage and processing. Currently, there are various low-cost EEG-based BCI devices available on the market [22]. However, many current models of EEG-based BCI become incommodious after continued use. Therefore, it is still necessary to improve their usability.

#### 2.1.1. Public Databases

Alternatively, there are also public databases with EEG data for affective information. Table 2 presents a list of available datasets related to emotion recognition. Such datasets are convenient for research, and several emotion recognition studies use them.

#### 2.1.2. Emotion Elicitation

The International Affective Picture System (IAPS) [31] and the International Affective Digitized Sound System (IADS) [32] are the most popular resources for emotion elicitation. These datasets provide emotional stimuli in a standardized way. Hence, it is useful for experimental investigations.

IAPS consists of 1200 images divided into 20 sets of 60 photos. Valence and arousal values are tagged for each photograph. IADS’ latest version provides 167 digitally recorded natural sounds familiar in daily life, with sounds labeled for valence, arousal, and dominance. Participants labeled the dataset using the Self-Assessment Manikin system [12]. IAPS and IADS stimuli are accessible with labeled information, which is convenient for the construction of a ground-truth for emotion assessment [33].

Other researchers used movie clips, which have also been shown capable of provoking emotions. In [34], the authors state that emotions using visual or auditory stimuli are similar. However, results obtained through affective labeling of multimedia may not be generalizable to more interactive situations or everyday circumstances. Thus, new studies using interactive emotional stimuli to ensure the generalizability of results for BCI would be welcomed.

Numerous experiments stimulated emotions in different settings, but they do not use EEG devices. However, they collected other physiological indicators as heartrate, skin galvanic changes, and respiration rate, among others. Conceptually, such paradigms could be useful if they are replicated for EEG signal acquisition. Possible experiments include stress during interviews for the detection of anger, anxiety, rejection, and depression. Exposure to odorants triggers emotions, such as anger, disgust, fear, happiness, sadness, and surprise. Harassment provokes fear. A threat of short-circuit, or a sudden backward-tilting chair elicits fear. A thread of shock provokes anxiety. Naturally, these EEG-based BCIs experiments should take into account ethical considerations.

To our knowledge, only a few studies have used more interactive conditions where participants played games or used flight simulators to induce emotions [35,36]. Alternatively, some authors have successfully used auto-induced emotions through memory recall [37].

#### 2.1.3. Normalization

EEG signals vary widely in amplitude depending on age, sex, and other factors like changes in subjects’ alertness during the day. Hence, it is necessary to normalize measured values to deal with this variability.

There are three possible approaches to normalization. The first is to record reference conditions without stimulus on the subject. The values obtained can be normalized by subtracting the reference value, then dividing by the reference value (or subtracting the reference value), and then dividing by that same value. The second approach also requires reference conditions. Those values are included in the feature vector, which will have twice the characteristics that make up the “baseline matrix”. The third approach normalizes the data separately by obtaining a specific range, for example, between −1 and 1. This method applied to each feature independently ensures that all characteristics have the same value ranges [38,39].

The effect of normalization and its influence on the entire process of emotion recognition is not yet evident. However, some studies show that normalization allows the characteristics to be generalized so that they can be used in cross-subject emotion recognition. Tangentially, data normalization helps machine learning algorithms’ efficiency due to faster convergence.

### 2.2. Preprocessing

EEG signals’ preprocessing relates to signal cleaning and enhancement. EEG signals are weak and easily contaminated with noise from internal and external sources. Thus, these processes are essential to avoid noise contamination that could affect posterior classification. The body itself may produce electrical impulses through blinking, eye or muscular movement, or even heartbeats that blend with EEG signals. It should be carefully considered whether these artifacts should be removed because they may have relevant emotional state information and could improve emotion recognition algorithms’ performance. If filters are used, it is necessary to use caution to apply them to avoid signal distortions.

The three commonly used filter types in EEG are (1) low-frequency filters, (2) high-frequency filters (commonly known by electrical engineers as low-pass and high-pass filters), and (3) notch filters. The first two filters are used to filter frequencies between 1 and 50–60 Hz. 

For EEG signal processing, filters, such as Butterworth, Chebyshev, or inverse Chebyshev, are preferred [39]. Each of them has specific features that need to be analyzed. A Butterworth filter has a flat response in the passband and the stopband but also has a wide transition zone. The Chebyshev filter has a ripple on the passband, and a steeper transition, so it is monotonic on the stopband. The inverse Chevishev has a flat response in the passband, is narrow in the transition, and has a ripple in the stopband. A Butterworth phase zero filter should be used to prevent a phase shift because this filter goes forward and backward over the signal to avoid this problem.

Another preprocessing objective is to clean the noise that may correspond to low-frequency signals generated by an external source, such as power line interference [40]. Notch filters are used to stop the passage of a specific frequency rather than a frequency range. This filter is designed to eliminate frequencies originated by electrical networks, and it typically ranges from 50 to 60 Hz depending on the electrical signal’s frequency in the specific country.

All of these filters are appropriate for artifact elimination in EEG signals. However, as previously noted, care must be taken when using filters. Generally, filters could distort the EEG signal’s waveform and structure in the time domain. Hence, filtering should be kept to a minimum to avoid loss of EEG signal information.

Nevertheless, preprocessing helps to separate different signals and sources. Table 3 shows methods used for preprocessing EEG signals [41] and the percentage in which they are mentioned in the literature as used from 2015 to 2020. Independent Component Analysis (ICA) and Principal Component Analysis (PCA) are tools that apply blind source analysis to isolate the source signal from noise when using multi-channel recordings so they can be used for artifact removal and noise reduction. Common Average Reference (CAR) is right for noise reduction. SL is applied for spatial filtering to improve the signal’s spatial resolution. The Common Spatial Patterns (CSP) algorithm finds spatial filters that could serve to distinguish signals corresponding to muscular movements.

Therefore, each of the most widely used preprocessing algorithms has its benefits. In Table 3, we can observe from the percentage of the usage column that the most utilized algorithms for preprocessing are PCA (50.1%), ICA (26.8%), and CSP (17.7%).

### 2.3. Feature Extraction

Once signals are noise free, the BCI needs to extract essential features, which will be fed to the classifier. Features can be computed in the domain of (1) time, (2) frequency, (3) time-frequency, or (4) space, as shown in Table 4 [31,38,39]. This table presents the most popular techniques used for feature extraction, their domain, advantages, and limitations.

Time-domain features include the event-related potential (ERP), Hjorth features, and higher-order crossing (HOC) [58,59,60], independent component analysis (ICA), principal component analysis (PCA), and Higuchi’s fractal dimensions (FD) as a measure of signal complexity and self-similarity in this domain. There are also statistical measures, such as power, mean, standard deviation, variance, skewness, kurtosis, relative band energy, and entropy. The latter evaluates signal randomness [61].

Among frequency-domain methods, the most popular is the fast Fourier transform (FFT). Auto-regressive (AR) modeling is an alternative to Fourier-based methods for computing the frequency spectrum of a signal [62,63].

The time-frequency domain exploits variations in time and frequency, which are very descriptive of the neural activities. For this, wavelet transform (WT) and wavelet packet decomposition (WPD) are used [62].

The spatial information provided in the description of EEG signals’ characteristics is also considered in a broader approach. For this dimension, signals are referenced to digitally linked ears (DLE) values, which are calculated in terms of the left and right earlobes as follows:

(1) $$V_{e}{}^{DLE}\;=\;V_{e}-\frac{1}{2}\left(V_{A1}+V_{A2}\right),$$

where *V_A_*_1_ and *V_A_*_2_ are the reference voltages on the left and right earlobe. Thus, EEG data is broken down, considering each electrode. Consequently, each channel contains spatial information of the location pertinent to its source.

For spatial computation, the surface Laplacian (SL) algorithm reduces volume conduction effects dramatically. SL also improves EEG spatial resolution by reducing the distortion produced by volume conduction and reference electrodes [47].

**Figure 4:**
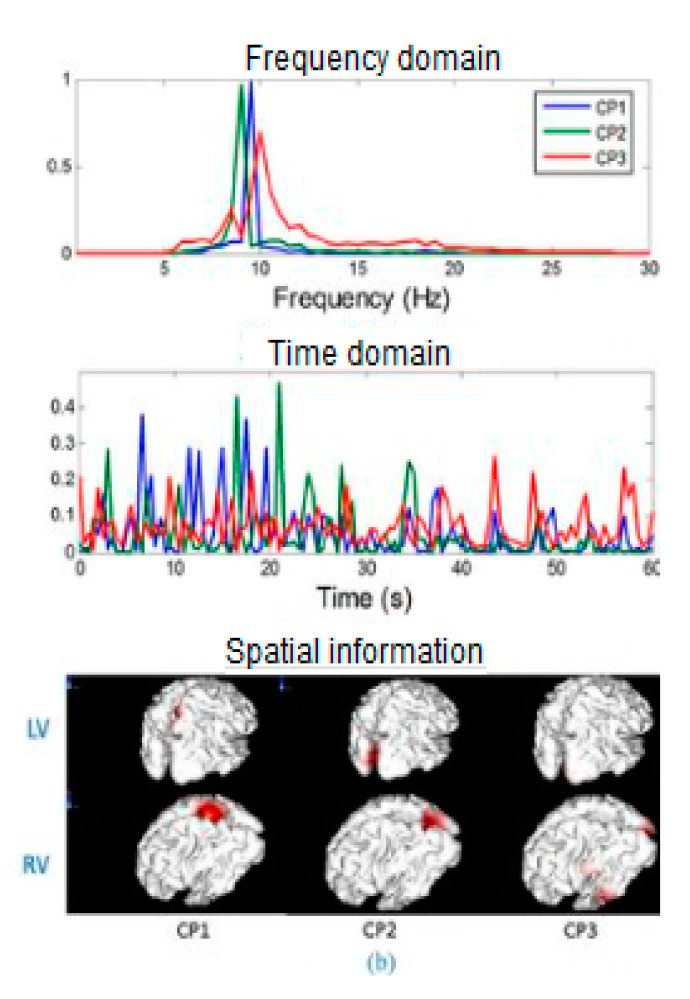
Frequency domain, time domain, and spatial information [63].

Figure 4 shows EEG signals in the time domain, the frequency domain, and spatial information.

According to [97], emotions emerge as the synchronization of various subsystems. Several authors use synchronized activity indexes in different parts of the brain. The efficiency of these indexes has been demonstrated in [98], calculating the correlation dimension of a group of EEG signals. In [98], other methods were used to calculate the synchronization of different areas of the brain. Synchronized indexes are a promising method for emotion recognition that deserves further research.

Table 4 shows the most commonly used algorithms and their respective mention percentages in the literature: (1) WT (26%), (2) PCA (19.7%), (3) Hjorth (17%), (4) ICA (11.3%), and (5) statistical measures (8.6%).

### 2.4. Feature Selection

The feature selection process is vital because it obtains the signal’s properties that best describe the EEG characteristics to be classified. In BCI systems, the feature vector generally has high dimensionality [99]. Feature selection reduces the number of input variables for the classifier (not to be confused with dimensionality reduction). While both processes decrease the data’s attributes, dimensionality reduction combines features to reduce their quantity.

A feature selection method does not change characteristics but excludes some according to specific usefulness criteria. Feature selection methods aim to achieve the best results by processing the least amount of data. It serves to remove attributes that do not contribute to the classification because they are irrelevant (or redundant) for simpler classification models (which are faster and have better performance). Additionally, feature selection methods reduce the overfitting likelihood in regular datasets, flexible models, or when the dataset has too many features but not enough observations.

One classification of feature selection methods based on the number of variables divides them into two classes: (1) Univariate and (2) multivariate. Univariate methods consider the input features one by one. Multivariate methods consider whole groups of characteristics together.

Another classification distinguishes feature selection methods as filtering, wrapper, and built-in algorithms.

- Filter methods evaluate features using the data’s intrinsic properties. Additionally, most of the filtering methods are univariate, so each feature is self-evaluated. These methods are appropriate for large data sets because they are less computationally expensive.
- Wrapping methods depend on classifier types when selecting new features based on their impact on characteristics already chosen. Only features that increase accuracy are selected.
- Built-in methods run internally in the classifier algorithms, such as deep learning. This type of process requires less computation than wrapper methods.

#### Examples of Feature Selection Algorithms

The following are some examples of algorithms for feature selection:

- Effect-size (ES)-based feature selection is a filter method.
  ES-based univariate: Cohen’s is an appropriate effect size for comparisons between two means [100]. So, if two groups’ means do not differ by 0.2 standard deviations or more, the difference is trivial, even if it is statistically significant. The effect size is calculated by taking the difference between the two groups and dividing it by the standard deviation of one of the groups. Univariate methods may discard features that could have provided useful information.
  ES-based multivariate helps remove several features with redundant information, therefore selecting fewer features, while retaining the most information [58]. It considers all the dependencies between characteristics when evaluating them. For example, calculating the Mahalanobis distance using the covariance structure of the noise.
  Min-redundancy max-relevance (mRMR) is a wrapper method [101]. This algorithm compares the mutual information between each feature with each class at the output. Mutual information between two random variables x and y is calculated as:
  (2) $$I\left(x;y\right)=\iint p\left(x,y\right)\log\frac{p\left(x,y\right)}{p\left(x\right)p\left(y\right)}dxdy,$$
  where *p* (*x*) and *p* (*y*) are the marginal probability density functions of *x* and *y*, respectively, and *p* (*x*, *y*) is their joint probability function. If *I* (*x*, *y*) equals zero, the two random variables x and y are statistically independent [58].
  mRMR maximizes *I* (*x_i_*, *y*) between each characteristic xi and the target vector y; and minimizes the average mutual information *I* (*x_i_*, *y_i_*) between two characteristics.
- Genetic algorithms allow the dimensionality of the feature vector to be reduced using evolutionary methods, leaving only more informative feature [2,86,97].
- Stepwise discriminant analysis SDA [74]. SDA is the extension of the statistical tool for discriminant analysis that includes the stepwise technique.
- Fisher score is a feature selection technique to calculate interrelation between output classes and each feature using statistic measures [101].

Table 5 shows feature selection algorithms and their percentage of usage in the literature. Genetic algorithms are frequently used (32.3%), followed by SDA (17.7%), wrapper methods (15.6%), and mRMR (11.5%).

### 2.5. Classification Algorithms

Model frameworks can categorize classification algorithms [56,57]. The model’s categories may be (1) generative-discriminative, (2) static-dynamic, (3) stable-unstable, and (4) regularized [102,103,104].

There are two different selection approaches for the classifier that works best under certain conditions in emotion recognition [56]. The first identifies the best classifier for a given BCI device. The second specifies the best classifier for a given set of features.

For synchronous BCIs, dynamic classifiers and ensemble combinations have shown better performances than SVMs. For asynchronous BCIs, the authors in this field have not determined an optimal classifier. However, it seems that dynamic classifiers perform better than static classifiers [56] because they handle better the identification of the onset of mental processes.

From the second approach, discriminative classifiers have been found to perform better than generative classifiers, principally in the presence of noise or outliers. Dynamic classifiers like SVM generally handle high dimensionality in the features better. If there is a small training set, simple techniques like LDA classifiers may yield satisfactory results [58].

#### 2.5.1. Generative Discriminative

These classifier models generally have supervised learning problems that fit the data’s probability. A generative model specifies the distribution of each class using the joint probability distribution p(x,y) and Bayes theorem. A discriminative model finds the decision boundary between the categories using the conditional probability distribution p(y|x). Such a model includes the following classifiers: Naïve Bayes, Bayesian networks, Markov random fields, and hidden Markov models (HMM).

#### 2.5.2. Static-Dynamic Classification

Static-dynamic classification takes into account the training method’s time variations. A static model trains the data once and then uses the trained model to classify a single feature vector. In a dynamic model, the system is updated continually. Thus, dynamic models can obtain a sequence of feature vectors and catch temporal dynamics.

Multilayer perceptron (MLP) can be considered a static classifier. Likewise, an example of a dynamic classifier is hidden Markov methods (HMM) because it can classify a sequence of feature vectors.

#### 2.5.3. Stable Unstable

Stable classifiers usually have low complexity and do not affect their performance with small variations of the training set. For example, k Nearest Neighbors (kNN) is a common stable classifier. Unstable classifiers have high complexity and present considerable changes in performance with minor variations of the training set. Examples of unstable classifiers are linear support vector machine (SVM), multi-layer perceptron (MLP), and bilinear recurrent neural network (BLR-NN).

#### 2.5.4. Regularized

Regularization consists of carefully controlling classifier complexity to prevent overtraining. These classifiers have excellent generalization performance. Regularized’s Fisher LDA (RF-LDA), linear SVM, and radial basis function kernel for support vector machine (RBF-SVM) are examples of regularized classifiers.

#### 2.5.5. General Taxonomy of Classification Algorithms 

Another taxonomy divides classifiers using their properties to distinguish them into general types of algorithms as linear, neural networks, nonlinear Bayesian, nearest neighbor classifiers, and combinations of systems (ensemble). Most of the more specialized algorithms can be generated from these general types. Table 6 shows this taxonomy criterion with five different categories of general classifiers: (1) Linear, (2) neural networks, (3) nonlinear Bayesian, (4) nearest neighbor classifiers, and (5) combinations of classifiers or ensemble [44,56,58].

All general classifiers have characteristics of each of the previously mentioned framework models. For instance, SVM is discriminant, static, stable, and regularized; HMM is generative, dynamic, unstable, and not regularized; and kNN is discriminant, static, stable, and not regularized. 

Consequently, the suggested guidelines for classifier selection are also applicable in this categorization. Table 6 presents the usage statistics of these classifiers in the 2015–2020 literature. The following are the most noteworthy classifiers: Neural networks CNN (46.16%), Linear classifiers SVM (30.3%), and LDA (5.5%), Nearest Neighbors kNN (4.5%), and Ensembled classifier AdaBoost (3.9%).

### 2.6. Performance Evaluation

Results must be reported consistently so that different research groups can understand and compare them. Hence, evaluation procedures need to be chosen and described accurately [119]. The evaluation of the classifier’s execution involves addressing performance measures, error estimation, and statistical significance testing [120]. Performance measures and error estimation configure the fulfillment rate of the classifier’s function. The most recommended performance evaluation measures are shown in Table 7. They are confusion matrix, accuracy, error rating, and other measures obtained from the confusion matrix, such as the recall, specificity, precision, Area Under the Curve (AUC), and F-measure. Other performance evaluation coefficients are Cohen’s kappa (k) [121], information transfer rate (ITR) [65], and written symbol rate (WSR) [121].

Performance evaluation and error estimation may need to be complemented with a significance evaluation. This is because high accuracies can be of little impact if the sample size is too small, or classes are imbalanced (labeled EEG signals typically are). Therefore, significance classification is essential. There are general approaches that can handle arbitrary class distributions to verify accuracy values that lie significantly above certain levels. Used methods are the theoretical level of random classification and adjusted Wald confidence interval for classification accuracy.

The theoretical level of random classification test classification results for randomness is the sum of the products between the experimental results’ classification probability and the probability calculated if all the categorization randomly occurs (p_0_ = classification accuracy of a random classifier). This approach can only be used after the classification has been performed [122].

Adjusted Wald confidence interval gives the lower and upper confidence limits for the probability of the correct classification, which specifies the intervals for the classifier performance evaluation index [123].

## 3. Literature Review of BCI Systems that Estimate Emotional States

In recent years, several research papers have been published in emotion recognition using BCI devices for data capture. Such publications use different models and strategies that produce a wide range of frameworks. Table 8 offers a summary of the research in this field from 2015 to 2020.

The following components characterize the systems presented in Table 8: (1) Stimulus type; (2) databases, generated by the paper’s authors or publicly available; (3) the number of participants; (4) extraction and selection of characteristics; (5) features; (6) classification algorithms; (7) number and types of classes; and (8) performance evaluation. 

The applied preprocessing methods are mostly similar in the reviewed studies. Their primary preprocessing methods are standard, so this information was omitted in Table 8.

### 3.1. Emotion Elicitation Methods

**Figure 5:**
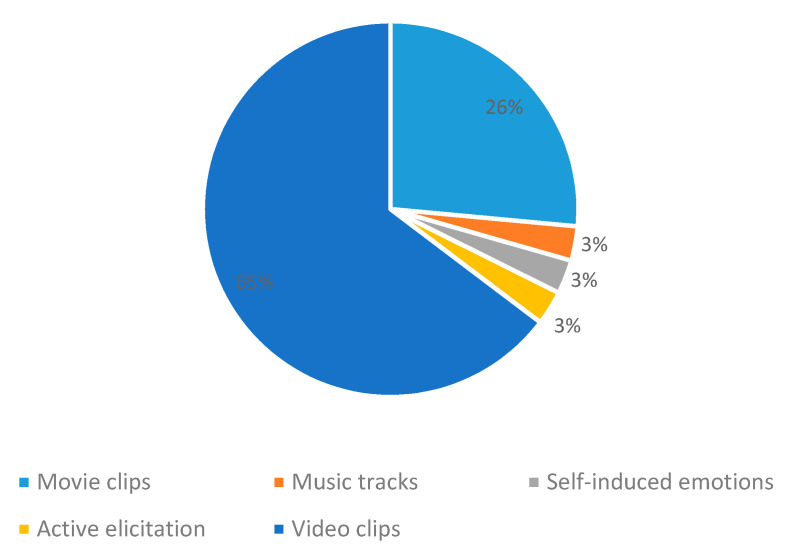
Emotion elicitation methods.

This article analyzes research papers that used different resources to provoke emotions in their subjects. These stimuli are music videos, film clips, music tracks, self-induced disgust (produced by remembering an unpleasant odor), and risky situations in a flight simulator as an example of active elicitation of emotions. EEG-based BCI systems frequently use the public DEAP and SEED databases that apply music videos and film clips as stimuli, respectively. Different stimuli provoke emotions that affect different areas of the brain and produce EEG signals that can be recognized concerning specific emotions. Figure 5 shows the frequency in which different emotion elicitation methods are applied to generate datasets used in the reviewed systems.

Few research papers resort to more elaborate platforms to provoke “real life” emotions. However, such methods have been applied to other physiological responses (other than EEG like skin conductance, respiration, electrocardiogram (ECG), facial expressions, among others) [124]. Some authors state that stimuli that provoke wide-ranging emotions could make it challenging to explore the brain’s mechanisms activated for specific emotion generation. In this sense, focusing on a particular emotion could improve our understanding of such mechanisms. For our research sample, we highlighted research pieces that study emotions, such as dislike, and disgust separately [37,125].

### 3.2. Number of Participants to Generate the System Dataset

**Figure 6:**
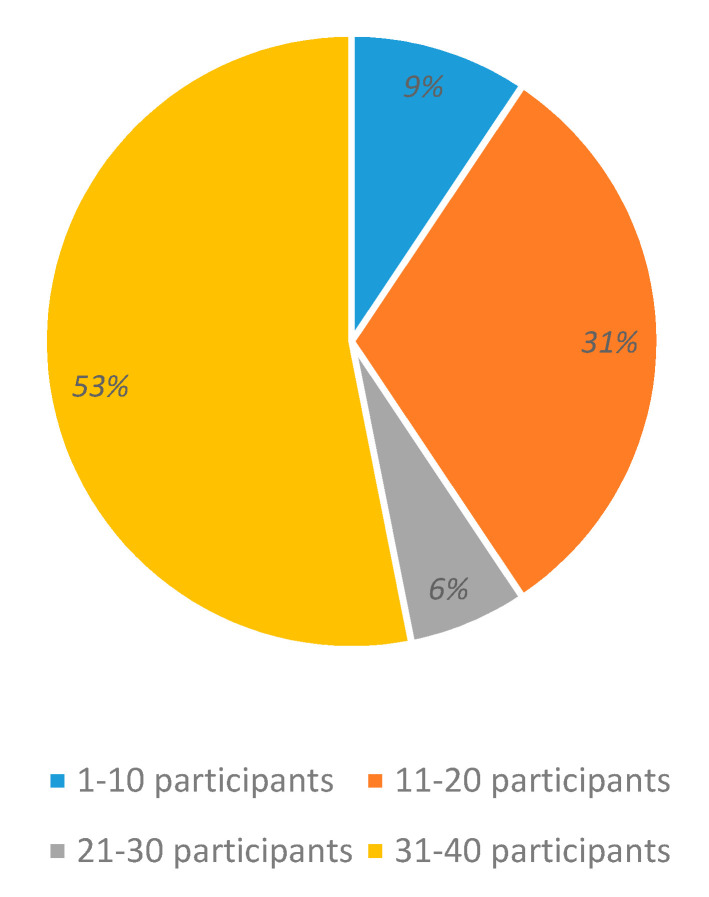
Number of participants in EEG datasets.

Figure 6 presents the number of participants in the experiments to obtain EEG datasets to train and test the emotion recognition systems. Most of the systems used a number of subjects in a range from 31–40 (53%), and 11–20 (31%). The targeted studies used EEG data from healthy individuals.

### 3.3. Datasets

**Figure 7:**
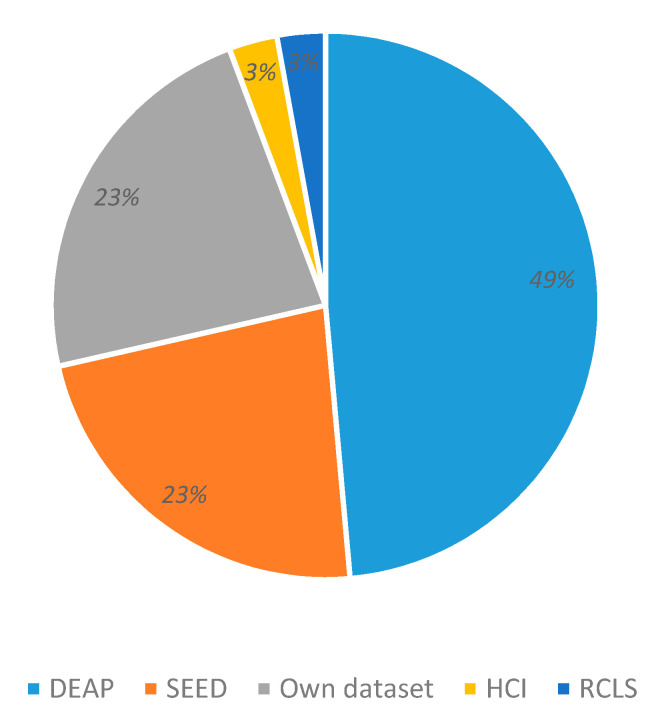
EEG datasets for emotion recognition.

Figure 7 presents the usage percentage of datasets used in emotion recognition. DEEP and SEED are publicly available databases, and are the most frequently used (49% and 23% of applications, respectively). Sometimes, other studies used self-generated datasets (23%), which are typically not freely accessible. The MAHNOB-HCI and RCLS public datasets appeared in our research sample, with a participation of 3% each.

Systems that use public databases offer some comparability, but contrast is limited even if the same characteristics are handled. Still, such public databases could eventually lead to findings if objective comparisons are performed.

### 3.4. Feature Extraction

Most systems use feature extraction methods in the time, frequency, time-frequency, or space domains. A small percentage of works evaluate the functional connectivity (or differences) in the observed activity between brain regions when emotions are provoked. Features with non-redundant information combined from different domains yield better classification results. However, it is still unclear if features work better alone or in combination with each other, or which type of features are more relevant for emotion recognition.

In our review, we found that researchers addressed these issues through the development of feature extraction algorithms that outperform the classic frequency bands and extract as much information as possible from brain signals. We believe that further developments should be connected to a comprehensive understanding of the brain’s neurophysiology.

**Figure 8:**
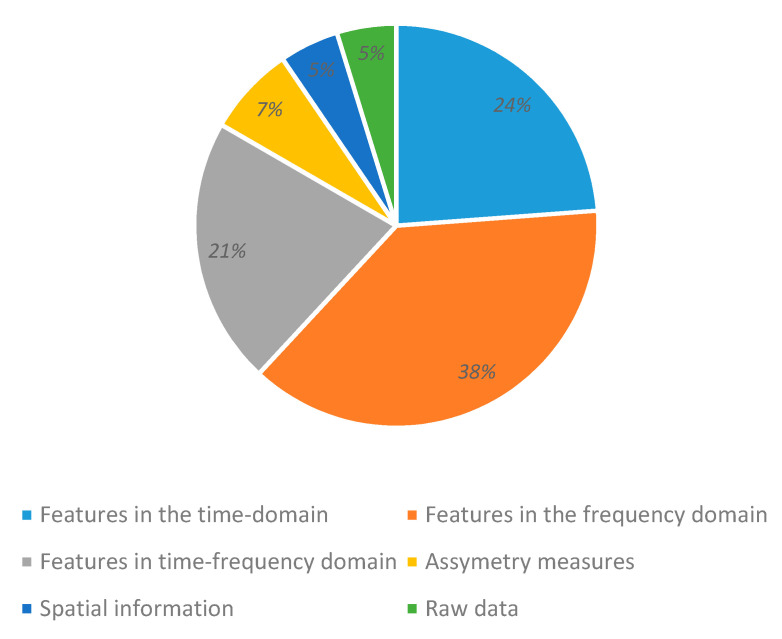
Domain of used features.

Figure 8 presents the domains of the used features. Frequency domain features are the most frequently used, and appear nearly twice as often as time domain or time-frequency domain features. Asymmetry characteristics between electrode pairs (by each hemisphere) are increasingly being used—likewise, electrodes’ location data in different brain sections. Additionally, raw data (without features) is used as inputs for deep learning classifiers.

**Figure 9:**
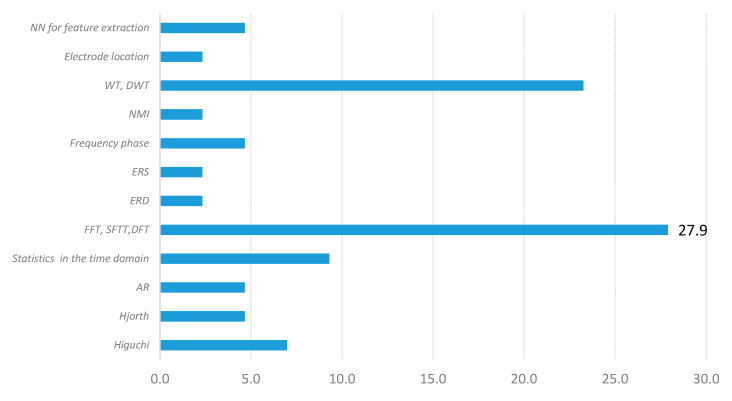
Percentage of the use of algorithms for feature extraction from Table 8.

Figure 9 shows the usage percentage of various algorithms for feature extraction computed in the 31 papers shown in Table 8. We found that FFT, SFFT, and DFT are the most commonly used tools for characteristic extraction in the frequency domain (27.9%). AR is used less frequently to estimate the spectrum (4.7%). WT and DWT appear in 23.3% of the systems in our sample. These algorithms are applied to obtain features in the time-frequency domain. Likewise, data from channel or electrode specific locations are less frequent (4.7%). Researchers also use statistics and computed parameters in the time domain (9.3%), normalized mutual information NMI (2.3%), ERS (2.3%), and ERD (2.3%).

We observed an increasing presence of algorithms embedded in neural networks like RBN, DBN, TensorFlow functions, and LSTM (4.7%) that are used to extract signal features automatically from raw data. This approach yields a good enough classifier performance, probably because it preserves information and avoids the risk of removing essential emotion-related signal features.

### 3.5. Feature Selection

It is worth noting that 61.3% of the systems presented in Table 8 do not use a feature selection method. Table 9 lists the systems that utilized feature selection algorithms. Interestingly, virtually every system uses a different algorithm except for the methods minimum redundancy maximum relevance (mRMR) and recursive feature elimination, which are utilized for two different schemes.

### 3.6. Classifiers

**Figure 10:**
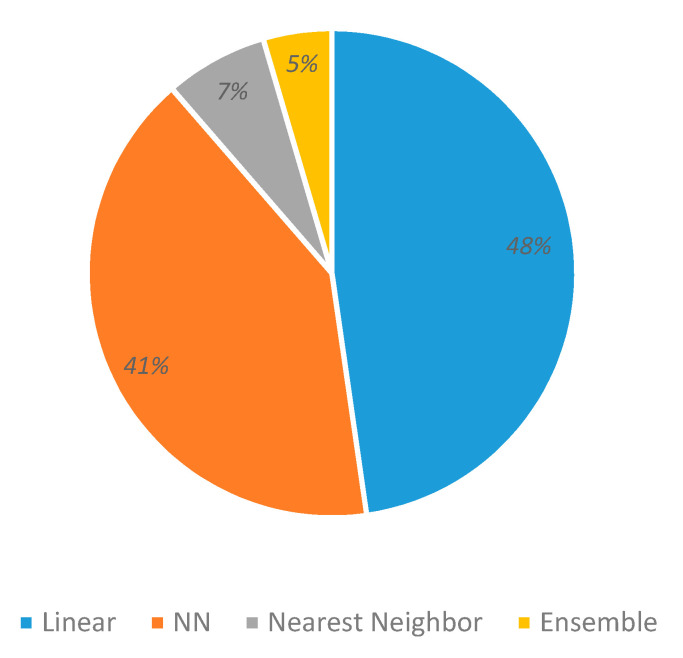
Classifiers’ usage.

Figure 10 shows that most classifiers were linear (48%) and neural networks (41%); a few papers used nearest neighbors (7%) and ensemble methods (5%). Consequently, it is worth mentioning that the following algorithms have become increasingly popular for EEG-based emotion recognition applications:

- Linear classifiers, such as naïve Bayes (NB), logistic regression (LR), support vector machine (SVM), linear discriminant analysis (LDA) (48% of use); and
- Neural networks like multi-layer perceptron (MLP), radial basis function RBF, convolutional neural network (CNN), deep belief networks (DBN), extreme learning method (ELM), graph regularized extreme learning machine (GELM), long short term memory (LSTM), domain adversarial neural network (DANN), Caps Net, and graph regularized sparse linear regularized (GRSLR) (41% of use).
- Ensemble classifiers like random forest, CART, bagging tree, Adaboost, and XGBoost are less used (5%). The same situation occurs with the kNN algorithm despite their consistently good performance results, probably because it works better with a simpler feature vector (7%).

During our considered period, this review did not find studies that applied non-linear Bayesian classifiers as hidden Markov models (HMM).

### 3.7. Performance vs. the Number of Classes-Emotions

The performance of almost all systems was evaluated using accuracy, except for two systems in which one used area under the curve (AUC), and the other one presented an F1 measure. Unfortunately, EEG datasets are usually unbalanced, with one or two labeled emotions more numerous than the others, which is somewhat problematic for this approach. Thus, this situation could lead to biased classifications. Moreover, EEG datasets are typically unbalanced, and performance measures should be calculated to contextualize their outcomes. In our view, this is why such results are not entirely comparable among different studies.

**Figure 11:**
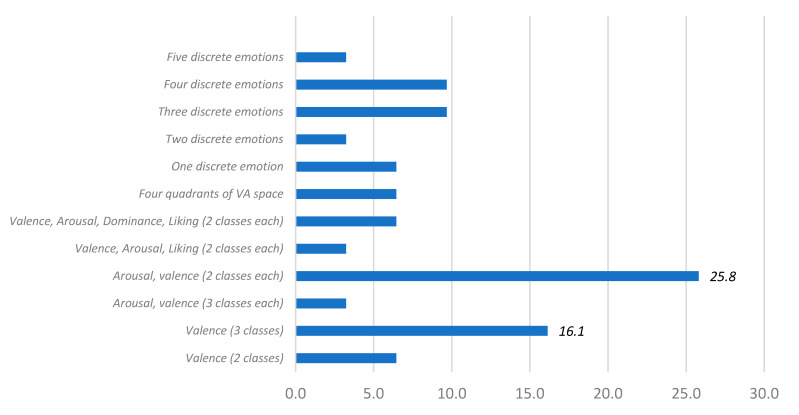
Percentage of systems with different numbers of classified emotions.

In Figure 11, we present the relationship between systems and the number of classified emotions. Most systems use the VA or VAD spaces and classify each dimension as a bi-class (for instance, valence positive and negative; arousal high-value and low value) or tri-class problem (for example, valence positive, neutral, and negative; arousal and dominance high-value and low-value).

Arousal and valence have the highest usage percentages (25.8%). On the other hand, 16.1% categorized valence with three classes: Positive, neutral, and negative. Then, 9.7% classified three discrete emotions like sadness, love, and anger. Moreover, lastly, 6.5% ranked valence as two classes (positive and negative), four discrete emotions (happy, sad, fear, and relaxed), one discrete emotion (disgust), or emotions located in one of four quadrants of the VA space (high valence-high arousal, high valence–low arousal, low valence–high arousal, and low valence–low arousal).

Classifier performance should be evaluated, taking into account that accuracy would be inversely proportional to the number of detected emotions. In other words, classification accuracy should be higher than a random classification process (equal chance for each class). Thus, as classification classes increase, a random classification process would yield a lower accuracy. For instance, a two-class random classification process would be 50% accurate. Likewise, three classes would imply a 33% classification accuracy for a random classification process, and so on. Therefore, such accuracy metrics should provide the classification performance benchmark for our evaluations.

**Figure 12:**
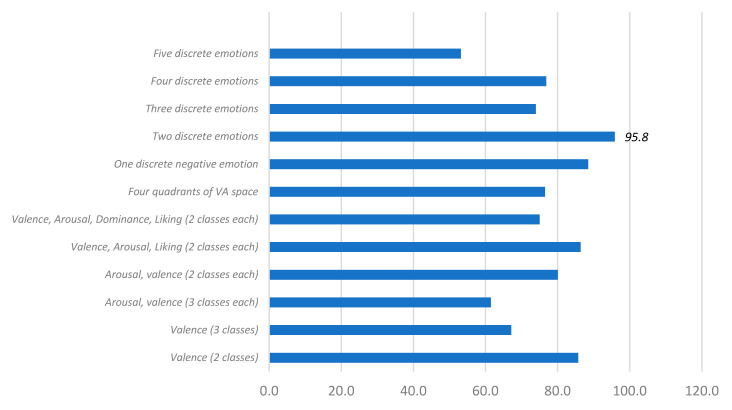
Accuracy vs. types and number of classified emotions.

Although the results of the performance of the systems depend on many factors, it is possible to find some relationship between the number of classes, the type of emotions classified, and the accuracy obtained (Figure 12). The best results are obtained with two classes, either as discrete emotions or as positive or negative values in a dimensional space. The second-best value is found for the recognition of one negative discrete emotion like dislike or disgust. The result that the classification of one emotion does not obtain the best performance value could be explained by the fact that in our review, we observed that negative emotions are more challenging to classify and tend to yield smaller performance values.

Comparing approaches and results obtained through different BCI-based systems is complex. This is because each system uses diverse experimental methods for emotion elicitation, protocols to detect EEG signals, datasets, extraction and selection of features, classification algorithms, and generally speaking, each implementation has different settings. Ideally, systems should be tested under similar conditions, but that scenario is not yet available. However, we can perform a comparative analysis to extract trends, bearing in mind such limitations. 

## 4. Future Work

Datasets developed for specific applications use passive methods to provoke emotions, such as IAPS, IADS, music videos, and film clips. Public databases, such as DEAP and SEED, use emotion elicitation through music videos and film clips, respectively. Few studies implement active emotion methods for provoking emotions, such as video games and flight simulators.

Going forward, we expect the generation of datasets that use active elicitation methods because these techniques simulate “real life” events better, and are more efficient at emotion induction. However, the implementation of such types of studies requires a significantly more complex experimental setup.

Furthermore, the study of individual emotions has been recently trending. Some works include fear detection, an analysis that has applications in phobia investigation, and other psychiatric disorders. It is worth mentioning that our survey found that negative emotions are more challenging to detect than positive ones.

We did not find in the literature the EEG-based emotion recognition of mixed feelings that combine positive and negative affects sensed at the same moment, for instance, bittersweet feelings. These mixed emotions are interesting because they are related to the study of higher creative performance [141].

Feature extraction and selection are EEG-based BCI system components, which are continuously evolving. They should be designed based on a profound understanding of the brain’s biology and physiology. The development of novel features is a topic that can contribute significantly to the improvement of results for emotion recognition systems. For instance, time-domain features are combined with frequency, time-frequency characteristics, channel location, and connectivity criteria. The development of novel feature extraction methods includes asymmetry discoveries in different functioning brain segments, new electrode locations that provide more information, connectivity models (between channels), and correlations needed for understanding functionality.

These evolving features contend that EEG signals and their frequency bands are related to multiple functional and connectivity considerations. The study of the relationship between EEG and biological or psycho-emotional elements should improve going forward. Improved features could better capture individual emotion dynamics and also correlate characteristics across individuals and sessions.

A particularly interesting trend in feature extraction is to use deep neural networks. These systems receive raw data to avoid loss of information and take advantage of the neural networks functioning to obtain relevant features automatically.

The overall reported system accuracy results range from 53% to 90% for the classification of one or more emotions. However, there likely is a gap between real-world applications performed in real time, which presents enormous challenges compared to experiments conducted in a laboratory. Some authors suggest that training datasets should be generated on a larger scale to overcome those challenges. Indeed, we believe it is reasonable that larger datasets could catalyze the research in this field. It is worth mentioning that a similar dynamic played out in the area of image recognition, which experienced a rapid expansion due to the generation of massive databases. Nevertheless, this effort for EEG datasets would likely require collaboration between various research groups to achieve emotions triggered by active elicitation methods.

Overall, we believe systems should be trained with larger sample sizes (and samples per subject), plus the use of real-time data. With such improved datasets, unsupervised techniques could be implemented to obtain comprehensive models. Moreover, these robust systems might allow for transfer learning, i.e., general models that can be applied successfully to particular individuals.

## 5. Conclusions

EEG signals are reliable information that cannot be simulated or faked. To decode EEG and relate these signals to specific emotion is a complex problem. Affective states do not have a simple mapping with specific brain structures because different emotions activate the same brain locations, or conversely, a single emotion can activate several structures.

In recent years, EEG-based BCI emotion recognition has been a field affecting computing that has generated much interest. Significant advances in the development of low-cost BCI devices with increasingly better usability have encouraged numerous research studies.

In this article, we reviewed the different algorithms and processes that can be part of EEG-based BCI emotion recognition systems: (1) Emotion elicitation, (2) signal acquisition, (3) feature extraction and selection, (4) classification techniques, and (5) performance evaluation. For our survey of this topic, we mined different databases and selected 60 studies carried out under a computer science perspective to gain insight into state of the art and suggest possible future research efforts.

As seen in this review, computational methods still do not have standards for various applications. Researchers continuing to look for new solutions in an ongoing effort. The study of the relationship between brain signals and emotions is a complex problem, and novel methods and new implementations are continuously presented. We expect that many of the existing challenges will soon be solved and will pave the way for a vast area of possible applications using EEG-based emotion recognition.

## Figures and Tables

**Table 1:** Frequency bands associations [16,17].

Band	State Association	Potential Localization	Stimuli
Gamma rhythm (above 30 HZ)	Positive valence. These waves are correlated with positive spiritual feelings. Arousal increases with high-intensity visual stimuli.	Different sensory and non-sensory cortical networks.	These waves appear stimulated by the attention, multi-sensory information, memory, and consciousness.
Beta (13 to 30 Hz)	They are related to visual self-induced positive and negative emotions. These waves are associated with alertness and problem-solving.	Motor cortex.	They are stimulated by motor activity, motor imagination, or tactile stimulation. Beta power increases during the tension of scalp muscles, which are also involved in frowning and smiling.
Alpha (8 to 13 Hz)	They are linked to relaxed and wakeful states, feelings of conscious awareness, and learning.	Parietal and occipital regions. Asymmetries reported: rightward-lateralization of frontal alpha power during positive emotions, compared to negative or withdrawal-related emotions, originates from leftward-lateralization of prefrontal structures.	These waves are believed to appear during relaxation periods with eyes shut while remaining still awake. They represent the visual cortex in a repose state. These waves slow down when falling asleep and accelerate when opening the eyes, moving, or even when thinking about the intention to move.
Theta (4 to 7 Hz)	They appear in relaxation states, and in those cases, they allow better concentration. These waves also correlate with anxious feelings.	The front central head region is associated with the hippocampal theta waves.	Theta oscillations are involved in memory encoding and retrieval. Additionally, individuals that experience higher emotional arousal in a reward situation reveal an increase of theta waves in their EEG [17]. Theta coma waves appear in patients with brain damage.
Delta (0 to 4 Hz)	They are present in deep NREM 3 sleep stage. Since adolescence, their presence during sleep declines with advancing age.	Frontal, temporal, and occipital regions.	Deep sleep. These waves also have been found in continuous attention tasks [18].

**Table 2:** Publicly available datasets.

Source	Dataset	Number of Channels	Emotion Elicitation	Number of Participants	Target Emotions
[19]	DEAP	32 EEG channels	Music videos	32	Valence, arousal, dominance, liking
[23]	eNTERFACE’06	54 EEG channels	Selected images from IAPS.	5	Calm, positive, exciting, negative exciting
[24]	headIT	-	Recall past emotions	31	Positive valence (joy, happiness) or of negative valence (sadness, anger)
[25]	SEED	62 channels	Film clips	15	Positive, negative, neutral
[26]	SEED-IV	62 channels	72 film clips	15	Happy, sad, neutral, fear
[27]	Mahnob-HCI-tagging	32 channels	Fragments of movies and pictures.	30	Valence and arousal rated with the self-assessment manikin
[28]	EEG Alpha Waves dataset	16 channels	Resting-state eyes open/closed experimental protocol	20	Relaxation
[29]	DREAMER	14 channels	Film clips	23	Rating 1 to 5 to valence, arousal, and dominance
[30]	RCLS	64 channels	Native Chinese Affective Video System	14	Happy, sad, and neutral

**Table 3:** Frequently used pre-preprocessing methods of EEG signals.

Preprocessing Method	Main Characteristics	Advantages	Limitations	Literature’s Usage Statistics % (2015–2020)
Independent component analysis (ICA) [42]	ICA separates artifacts from EEG signals into independent components based on the data’s characteristics without relying on reference channels. It decomposes the multi-channel EEG data into temporal separate and spatial-fixed components. It has been applied for ocular artifact extraction.	ICA efficiently separates artifacts from noise components. ICA decomposes signals into temporal independent and spatially fixed components.	ICA is successful only under specific conditions where one of the signals is of greater magnitude than the others. The quality of the corrected signals depends strongly on the quality of the artifacts.	26.8
Common Average Reference (CAR) [43,44]	CAR is used to generate a reference for each channel. The algorithm obtains an average or all the recordings on every electrode and then uses it as a reference. The result is an improvement in the quality of Signal to Noise Ratio.	CAR outperforms standard types of electrical referencing, reducing noise by >30%.	The average calculation may present problems for finite sample density and incomplete head coverage.	5.0
Surface Laplacian (SL) [45,46,47,48,49]	SL is a way of viewing the EEG data with high spatial resolution. It is an estimate of current density entering or leaving the scalp through the skull, considering the volume conductor’s outer shape and does not require details of volume conduction.	SL estimates are reference-free, meaning that any EEG recording reference scheme will render the same SL estimates. SL enhances the spatial resolution of the EEG signal. SL does not require any additional assumptions about functional neuroanatomy.	It is sensitive to artifacts and spline patterns.	0.4
Principal Component Analysis (PCA) [35,50,51,52,53,54,55]	PCA finds patterns in data. It can be pictured as a rotation of the coordinate axes so that they are not along with single time points. Still, along with linear combinations of sets of time points, collectively represents a pattern within the signal. PCA rotates the axes to maximize the variance within the data along the first axis, maintaining their orthogonality.	PCA helps in the reduction of feature dimensions. The ranking will be done and helps in the classification of data.	PCA does not eliminate noise, but it can reduce it. PCA compresses data compared to ICA and allows for data separation.	50.1
Common Spatial Patterns (CSP) [55,56,57]	CSP applies spatial filters that are used to discriminate different classes of EEG signals. For instance, those corresponding to different motor activity types. CSP also estimates covariance matrices.	CSP does not require a priori selection of sub-specific bands and knowledge of these bands.	CSP requires many electrodes. Changes in electrode location may affect classification accuracies.	17.7

**Table 4:** Feature extraction algorithms.

Feature Extraction Method	Main Characteristics	Domain	Advantages	Limitations	Literature’s usage statistics % (2015–2020)
ERP [18,40,64,65,66,67,68,69]	It is the brain response to a sensory, cognitive, or motor event. Two sub-classifications are (1) evoked potentials and (2) induced potentials.	Time	It has an excellent temporal resolution. ERPs provide a measure of the processing between a stimulus and a response.	ERP has a poor spatial resolution, so it is not useful for research questions related to the activity location.	2.9
Hjorth Features [52,59,60]	These are statistical indicators whose parameters are normalized slope descriptors. These indicators are activity (variance of a time function), mobility (mean frequency of the proportion of standard deviation of the power spectrum), and complexity (change in frequency compared to the signal’s similarity to a pure sine wave).	Time	Low computational cost appropriate for real-time analysis.	Possible statistical bias in signal parameter calculations	17.0
Statistical Measures [39,40,42,52,61,62,63,64,65,66,67,68,69,70]	Signal statistics: power, mean, standard deviation, variance, kurtosis, relative band energy.	Time	Low computational cost.	-	8.6
DE [1,10,11,15,59,68,71,72,73,74,75,76,77,78,79,80,81,82,83,84]	Entropy evidences scattering in data. Differential Entropy can reflect spatial signal variations.	Time–spatial	Entropy and derivate indexes reflect the intra-cortical information flow.		4.9
HOC [1,2,42,63,85,86,87,88]	Oscillation in times series can be represented by counts of axis crossing and its differences. HOC displays a monotone property whose rate of increase discriminates between processes.	Time	HOC reveals the oscillatory pattern of the EEG signal providing a feature set that conveys enough emotion information to the classification space.	The training process is time-consuming due to the dependence of the HOC order on different channels and different channel combinations [60].	2.0
ICA [20,37,53,69,89,90,91]	ICA is a signal enhancing method and a feature extraction algorithm. ICA separates components that are independent of each other based on the statistical independence principle.	Time. There is also a FastICA in the frequency domain.	ICA efficiently separates artifacts from noise components. ICA decomposes signals into temporal independent and spatially fixed components.	ICA is only useful under specific conditions (one of the signals is of greater magnitude than the others). The quality of the corrected signals depends strongly on the quality of the isolated artifacts.	11.3
PCA [33,40,52,69,92,93,94,95]	The PCA algorithm is mostly used for feature extraction but could also be used for feature extraction. It reduces the dimensionality of the signals creating new uncorrelated variables.	Time	PCA reduces data dimensionality without information loss.	PCA assumes that the data is linear and continuous.	19.7
WT [48]	The WT method represents the original EEG signal with secured and straightforward building blocks known as wavelets, which can be discrete or continuous.	Time-frequency	WT describes the features of the signal within a specified frequency domain and localized time domain properties. It is used to analyze irregular data patterns. Uses variable windows, wide for low frequencies, and narrow for high frequencies.	High computational and memory requirements.	26.0
AR [48]	AR is used for feature extraction in the frequency domain. AR estimates the power spectrum density (PSD) of the EEG using a parametric approach. The estimation of PSD is achieved by calculating the coefficients or parameters of the linear system under consideration.	Frequency domain	AR is used for feature extraction in the frequency domain. AR limits the leakage problem in the spectral domain and improves frequency resolution.	The order of the model in the spectral estimation is challenging to select. It is susceptible to biases and variability.	1.6
WPD [95]	WPD generates a sub-band tree structuring since a full binary tree can characterize the decomposition process. WPD decomposes the original signals orthogonally and independently from each other and satisfies the law of conservation of energy. The energy distribution is extracted as the feature.	Time-frequency	WPD can analyze non-stationary signals such as EEG.	WPD uses a high computational time to analyze the signals.	1.6
FFT [48]	FFT is an analysis method in the frequency domain. EEG signal characteristics are reviewed and computed by power spectral density (PSD) estimation to represent the EEG samples signal selectively.	Frequency	FFT has a higher speed than all the available methods so that it can be used for real-time applications. It is a useful tool for stationary signal processing.	FFT has low-frequency resolution and high spectral loss of information, which makes it hard to find the actual frequency of the signal.	2.2
Functional EEG connectivity indices [15]	EEG-based functional connectivity is estimated in the frequency bands for all pairs of electrodes using correlation, coherence, and phase synchronization index. Repeated measures of variance for each frequency band were used to determine different connectivity indices among all pairs.	Frequency	Connectivity indices at each frequency band can be used as features to recognize emotional states.	Difficult to generalize and distinguish individual differences in functional brain activity.	1.3
Rhythm [14,56]	Detection of repeating patterns in the frequency band or “rhythm”.	Frequency	Specific band rhythms contribute to emotion recognition.	-	0.1
Graph Regularized Sparse Linear Regularized GRSLR [30]	This method applies a graph regularization and a sparse regularization on the transform matrix of linear regression	Frequency	It can simultaneously cope with sparse transform matrix learning while preserving the intrinsic manifold of the data samples.	-	0.2
Granger causality [63,96]	This feature is a statistical concept of causation that is based on prediction.	Frequency	The authors can analyze the brain’s underlying structural connectivity.	These features only give information about the linear characteristics of signals.	0.6

**Table 5:** Feature selection methods used in the literature (2015–2020) in percentages (%).

Feature Selection Method	Literature’s Usage Statistics % (2015–2020)
min-Redundancy Max-Relevance mRMR	11.5%
Univariate	6.3%
Multivariate	6.3%
Genetic Algorithms	32.3%
Stepwise Discriminant Analysis SDA	17.7%
Fisher score	7.3%
Wrapper methods	15.6%
Built-in methods	3.1%

**Table 6:** Categories of general classifiers.

Category of Classifier	Description	Examples of Algorithms in the Category	Advantages	Limitations	Literature’s Usage Statistics % (2015–2020)
Linear	Discriminant algorithms that use linear functions (hyperplanes) to separate classes.	Linear Discriminant Analysis LDA [65]. Bayesian Linear Discriminant Analysis. Support Vector Machine SVM [105,106]. Graph Regularized Sparse Linear Regularized GRSLR [30].	These algorithms have reasonable classification accuracy and generalization properties.	Linear algorithms tend to have poor outcomes in processing complex nonlinear EEG data.	5.50 1.40 30.30 0.02
Neural networks (NN)	NN are discriminant algorithms that recognize underlying relationships in a set of data resembling the human brain operation.	Multilayer Perceptron MLP [107]. Long Short-term Memory Recurrent Neural Network LSTM-RNN [66,67,68,69]. Domain Adversarial Neural Network DANN [108]. Convolutional Neural Network CNN [68,70,71,72,73,109,110,111]. Complex-Valued Convolutional Neural Network CVCNN [105]. Gated-Shape Convolutional Neural Network GSCNN [105]. Global Space Local Time Filter Convolutional Neural Network GSLTFCNN [105]. CapsNet-NN Genetic Extreme Learning Machine GELM–NN [82].	NN generally yields good classification accuracy	Sensitive to overfitting with noisy and non-stationary data as EEGs.	1.60 1.10 0.20 46.16 0.40 0.40 0.02 0.10 0.10
Nonlinear Bayesian classifier	Generative classifiers produce nonlinear decision boundaries.	Bayes quadratic [110]. Hidden Markov Model HMM [50,112].	Generative classifiers reject uncertain samples efficiently.	For Bayes quadratic, the covariance matrix cannot be estimated accurately if the dimensionality is vast, and there are not enough training sample patterns.	0.10 0.30
Nearest neighbor classifiers	Discriminative algorithms that classify cases based on its similarity to other samples	k-Nearest Neighbors kNN [113]. Mahalanobis Distance [114].	kNN has excellent performance with low-dimensional feature vectors. Mahalanobis Distance is a simple but efficient classifier, suitable even for asynchronous BCI.	kNN has reduced performance for classifying high dimension feature vectors or noise distorted features.	4.5 0.1
Combination of classifiers (ensemble-learning)	Combined classifiers using boosting, voting, or stacking. Boosting consists of several cascading classifiers. In voting, classifiers have scores, which yield a combined score per class, and a final class label. Stacking uses classifiers as meta-classifier inputs.	Ensemble-methods can combine almost any type of classifier [115]. Random Forest [10,116]. Bagging Tree [111,115]. XGBoost [117] AdaBoost [118]	Variance reduction that leads to increase of classification accuracy.	Quality measures are application dependent.	2.1 1.1 0.2 0.4 3.9

**Table 7:** Conventional performance evaluation methods for BCI.

Performance Evaluation	Main characteristics	Advantages	Limitations
Confusion matrix	The confusion matrix presents the number of correct and erroneous classifications specifying the erroneously categorized class.	The confusion matrix gives insights into the classifier’s error types (correct and incorrect predictions for each class). It is a good option for reporting results in M-class classification.	Results are difficult to compare and discuss. Instead, some authors use some parameters extracted from the confusion matrix.
Accuracy and error rate	The accuracy p is the probability of correct classification in a certain number of repeated measures. The error rate is e = 1 − p and corresponds to the probability that an incorrect classification has been made.	It works well if the classes are balanced, i.e., there are an equal number of samples belonging to each class.	Accuracy and error rate do not take into account whether the dataset is balanced or not. If one class occurs more than another, the evaluation may appear with a high value for accuracy even though the classification is not performing well. These parameters depend on the number of classes and the number of cases. In a 2-class problem the chance level is 50%, but with a confidence level depending on the number of cases.
Cohen’s kappa (k)	k is agreement evaluation between nominal scales. This index measures the agreement between a true class compared to a classifier output. 1 is a perfect agreement, and 0 is pure chance agreement.	Cohen’s kappa returns the theoretical chance level of a classifier. This index evaluates the classifier realistically. If k has a low value, the confusion matrix would not have a meaningful classification even with high accuracy values. This coefficient presents more information than simple percentages because it uses the entire confusion matrix.	This coefficient has to be interpreted appropriately. It is necessary to report the bias and prevalence of the k value and test the significance for a minimum acceptable level of agreement.
Sensitivity or Recall	Sensitivity, also called Recall, identifies the true positive rate for describing the accuracy of classification results. It evaluates the proportion of correctly identified true positives related to the sum of true positives plus false negatives.	Sensitivity measures how often a classifier correctly categorizes a positive result.	The Recall should not be used when the positive class is larger (imbalanced dataset), and correct detection of positives samples is less critical to the problem.
Specificity	Specificity is the ability to identify a true negative rate. It measures the proportion of correctly identified true negatives over the sum of the true negatives plus false positives. The False Positive Rate (FPR) is then equal to 1 – Specificity.	Specificity measures how often a classifier correctly categorizes a negative result.	Specificity focuses on one class only, and the majority class biases it.
Precision	Precision also referred to as Positive Predicted Value, is calculated as 1 – False Detection Rate (F). False detection rate is the ratio between false positives over the sum of true positives plus false positives.	Precision measures the fraction of correct classifications.	Precision should not be used when the positive class is larger (imbalanced dataset), and correct detection of positives samples is less critical to the problem.
ROC	The ROC curve is a Sensitivity plot as a function of the False Positive Rate. The area under the ROC curve is a measure of how well a parameter can distinguish between a true positive and a true negative.	ROC curve provides a measure of the classifier performance across different significance levels.	ROC is not recommended when the negative class is smaller but more important. The Precision and Recall will mostly reflect the ability to predict the positive class if it is larger in an imbalanced dataset.
F-Measure	F-Measure is the harmonic mean of Precision and Recall. It is useful because as the Precision increases, Recall decreases, and vice versa.	F-measure can handle imbalanced data. F-measure (like ROC and kappa) provides a measure of the classifier performance across different significance levels.	F-measure does not generally take into account true negatives. True negatives can change without affecting the F-measure.
Pearson correlation coefficient	Pearson’s correlation coefficient (r), quantifies the degree of a ratio between the true and predicted values by a value ranking from −1 to +1.	Pearson’s correlation is a valid way to measure the performance of a regression algorithm.	Pearson’s correlation ignores any bias which might exist between the true and the predicted values.
Information transfer rate (ITR)	As BCI is a channel from the brain to a device, it is possible to estimate the bits transmitted from the brain. ITR is a standard metric for measuring the information sent within a given time in bits per second.	ITR is a metric that contributes to criteria to evaluate a BCI System.	ITR is often misreported due to inadequate understanding of many considerations as delays are necessary to process data, to present feedback, and clear the screen. TR is best suited for synchronous BCIs over user-paced BCI.

**Table 8:** Summary of emotion recognition systems using BCI ^1^.

Reference/Year	Stimuli	EEG Data	Feature Extraction	Feature Selection	Features	Classification	Emotions	Accuracy
[126]/2016	-	DEAP	Computation in the time domain, Hjorth, Higuchi, FFT	mRMR	Statistical features, BP, Hjorth, FD	RBF NN SVM	3 class/Arousal 3 class/Valence	Arousal/60.7% Valence/62.33%
[85]/2015	15 movie clips	Own dataset/15 participants	DBN	-	DE, DASM, RASM, DCAU, from Delta, Theta, Alpha, Beta, and Gamma.	kNN LR SVM DBNs	Positive Neutral Negative.	SVM/83.99% DBN/86.08%
[37]/2015	Self-induced emotions	Own dataset/10 participants	WT	PCA	Eigenvalues vector	SVM	Disgust	Avg. 90.2%
[127]/2018	Video clips	Own dataset/10 participants	Higuchi	-	FD	RBF SVM	Happy Calm Angry	Avg. 60%
[128]/2017	Video clips	Own dataset/30 participants	SFTT, ERD, ERS	LDA	PSD	LIBSVM	Joy Amusement Tenderness Anger Disgust Fear Sadness Neutrality	Neutrality 81.26% 3 Positive emotions 86.43% 4 Negative emotions 65.09%
[125]/2020	-	DEAP	DFT, DWT	-	PSD, Logarithmic compression of Power Bands, LFCC, PSD, DW	NB CART kNN RBF SVM SMO	Dislike	Avg. SMO/81.1% NB/63.55% kNN/86.73% CAR/74.08%
[86]/2019	-	DEAP and SEED-IV	Computations in time domain, FFT, DWT	-	PSD, Energy, DE, Statistical features	SVM	HAHV HALV LALV LAHV	Avg DEAP/79% Avg.SEED/76.5%
[14]/2016	Music tracks	Own dataset/30 participants	SFTT, WT	-	PSD, BP Entropy, Energy, Statistical features, Wavelets	SVM MLP kNN	Happy Sad Love Anger	Avg. SVM/75.62% MLP/78.11% kNN/72.81%
[79]/2017	-	SEED	FFT, and electrode location	Max Pooling	DE, DASM, RASM, DCAU	SVM ELM Own NN method	Positive Negative Neutral	Avg. SVM/74.59% ELM/74.37% Own NN/86.71%
[48]/2019	Video clips	Own dataset/16 participants	SFTT, WT, Hjorth, AR	-	PSD, BP, Quadratic mean, AR Parameters, Hjorth	SVM	Happy Sad Fear Relaxed	Avg. 90.41%
[129]/2019	-	DEAP	WT	-	Wavelets	LSTM RNN	Valence Arousal	Avg. 59.03%
[130]/2018	-	SEED	LSTM to learn context information for each hemispheric data	-	DE	BiDANN	Positive Negative Neutral	Avg. 92.38%
[111]/2019	-	DEAP	Signal computation in the time domain, and FFT		Statistical characteristics. PSD	BT SVM LDA BLDA CNN	Valence Arousal	Avg. for combination features AUC BT/0.9254 BLDA/0.8093 SVM/0.7460 LDA/0.5147 CVCNN/0.9997 GSCNN/1 GSCNN/1
[118]/2017	-	DEAP	Computation in the time domain, and FFT	GA	Statistical characteristics, PSD, and nonlinear dynamic characteristics	AdaBoost	Joy Sadness	95.84%
[131]/2019	-	DEAP	SFTT, NMI	-	Inter-channel connection matrix based on NMI	SVM	HAHV HALV LALV LAHV	Arousal/73.64% Valence/74.41%
[74]/2018	-	SEED	FFT	SDA	Delta, Theta, Alpha, Beta, and, Gamma	LDA	Positive Negative Neutral	Avg. 93.21%
[112]/2019	-	SEED	FFT	-	Electrodes-frequency Distribution Maps (EFDMs)	CNN	Positive Negative Neutral	Avg. 82.16%
[80]/2019	-	SEED/ DEAP/ MAHNOB-HCI	Computation in the time domain, and FFT	Fisher-score, classifier-dependent structure (wrapper), mRMR, SFEW	EEG based network patterns (ENP) PSD, DE, ASM, DASM, RASM, DACU, ENP, PSD + ENP, DE + ENP	SVM GELM	Positive Negative Neutral	Best feature F1 SEED/DE+ENP gamma 0.88 DEAP/PSD+ENP gamma 0.62 MAHNOB/PSD+ENP Gamma 0.68
[96]/2019	-	DEAP	Tensorflow framework	Sparse group lasso	Granger causality feature	CapsNet Neural Network	Valence-arousal	Arousal/87.37% Valence/88.09%
[30]/2019	Video clips	Own dataset RCLS/14 participants. SEED	Computation in the time domain, WT	-	HOC, FD, Statistics, Hjorth, Wavelets	GRSLR	Happy Sad Neutral	81.13%
[132]/2019	-	DEAP	Computation in the time domain, FFT, WT	Correlation matrix, information gain, and sequential feature elimination	Statistical measures, Hjorth, Autoregressive parameters, frequency bands, the ratio between frequency bands, wavelet domain features	XGBoost	Valence, arousal, dominance, and liking	Valence/75.97% Arousal/74.20% Dominance/75.23% Liking 76.42%
[133]/2015	-	DEAP	Frequency phase information	Sequential feature elimination	Derived features of bispectrum	SVM	Low/high valence, low/high arousal	Low-high arousal/64.84% Low-high valence/61.17%
[134]/2016	-	DEAP	Higuchi, FFT	-	FD, PSD	SVM	Valence, arousal	Valence/86.91% Arousal/87.70%
[135]/2017	-	DEAP	DWT	-	Discrete wavelets	kNN	Valence, arousal	Valence/84.05% Arousal/86.75%
[136]/2015	-	DEAP	RBM	-	Raw signal-6 channels	Deep-Learning	Happy, calm, sad, scared	Avg. 75%
[137]/2017	-	DEAP	DWT	Best classification performance for channel selection	Discrete wavelets	MLP kNN	Positive, negative	MLP/77.14% kNN/72.92%
[138]/2017	-	DEAP	-	-	-	LSTM NN	Low/high valence, Low/high arousal, Low/high liking	Low-high valence/85.45% Low-high arousal/85.65% Low-high liking/87.99%
[139]/2018	-	DEAP	-	-	-	3D-CNN	Valence, arousal	Valence/87.44% Arousal/88.49%
[140]/2018	-	DEAP	FFT, phase computations, Pearson correlation	-	PSD, phase, phase synchronization, Pearson correlation	CNN	Valence	Valence/96.41%
[36]/2019	Flight simulator	Own dataset/8 participants	Computation in time domain, and WT	-	Statistical measures, DE, Wavelets	ANN	Happy, Sad, Angry, Surprise, Scared	Avg. 53.18%

^1^ Autoregressive Parameter (AR). Bagging Tree (BT). Band Power (BP). Bayesian linear discriminant analysis (BLDA). Bi-hemispheres Domain Adversarial Neural Network (BiDANN). Convolutional Neural Network (CNN). Complex-Valued Convolutional Neural Network (CVCNN). Gated-Shape Convolutional Neural Network (GSCNN). Global Space Local Time Filter Convolutional Neural Network (GSLTFCNN). Deep Belief Networks (DBNs). Differential entropy (DE). DE feature Differential Asymmetry (DASM). DE feature Rational Assimetry (RASM). DE feature Differential Caudality (DCAU). Electrooculography (EOG). Electromyogram (EMG). Event-Related Desynchronization (ERD) and Synchronization (ERS). Feature selection and weighting method (SFEW). Fractal dimensions (FD). Genetic Algorithm (GA). Graph regularized Extreme Learning Machine (GELM) NN. Graph Regularized Sparse Linear Regularized (GRSLR). High Order Crossing (HOC). Linear Discriminant Analysis (LDA). Logistic Regression (LR). Long short-term memory Recurrent Neural Network (LSTM RNN). Minimum-Redundancy-Maximum-Relevance (mRMR). Normalized Mutual Information (NMI). Principal Component Analysis (PCA). Radial Basis Function (RBF). Short-Time Fourier Transform (STFT). Stepwise Discriminant Analysis (SDA). Support Vector Machine (SVM). Wavelet Transform (WT).

**Table 9:** Systems in Table 8 using feature selection algorithms.

Feature Selection Algorithm	Reference
mRMR	[80,126]
PCA	[38]
LDA	[128]
Max Pooling	[79]
Genetic Algorithm	[118]
SDA	[75]
Fisher-score	[80]
SFEW	[80]
Sparse group lasso	[96]
Correlation matrix	[132]
Information gain	[132]
Recursive feature elimination	[132,133]
Best classification performance for channel selection	[137]
